# Supplementary material for: Are carbon emissions trading and green financial instruments synergistic? -Comprehensive quantitative research based on content analysis
Source: PLoS One. 2024 Mar 7;19(3):e0298601. doi: 10.1371/journal.pone.0298601 (PMC10919601; doi:10.1371/journal.pone.0298601)
Supplement: S1 Data — (ZIP) [file pone.0298601.s001.zip › Data/地级市Z_F工作报告.zip!/╔╜╢1⁄2/╚╒╒╒╩╨/└┤╫╘╣1⁄2╓┌║┼í╛┬φ┐╦╩2╛▌═°í┐_2009.docx]

各位代表：

现在，我代表市人民政府向大会作政府工作报告，请予审议，并请市政协各位委员和列席会议的其他同志提出意见。

一、2008年政府工作回顾

刚刚过去的2008年是很不寻常、很不平凡的一年，大事多，难事多，任务重，要求高。我们经受了物价上涨、煤电油运紧张、人民币升值、抗震救灾、清理浒苔、处置“问题奶粉”以及国际金融危机冲击等一系列难以预料、历史罕见的重大挑战和考验。面对复杂多变的形势和艰巨繁重的任务，市政府紧紧依靠省委、省政府和市委的坚强领导，依靠市人大、市政协的监督支持，依靠全市人民的共同努力，坚持以科学发展观为统领，坚持“一切为了发展，一切为了人民”，深入实施“四大战略”和“双轮驱动”，全力推动经济社会科学发展、跨越发展、和谐发展，圆满完成了市十六届人大一次、二次会议确定的各项任务，主要经济指标增幅位居全省前列，一批事关长远和大局的项目取得重大进展，新一届市政府各项工作实现了良好开局。初步统计，2008年全市生产总值达到780亿元，比上年增长15.5%，增幅高于全省平均水平。实现境内财政总收入218.66亿元，增长46.1%，其中地方财政收入36.33亿元，增长25.4%，增幅居全省首位。完成规模以上固定资产投资518.43亿元，增长26.4%。实现社会消费品零售总额212.36亿元，增长23.3%。进出口总值首次进入全省四强，达到92.68亿美元，增长60.9%，其中出口25.25亿美元，增长28.6%。城镇居民人均可支配收入14409元，农民人均纯收入6000元，分别增长10.7%和12%。

（一）积极应对接踵而至的困难和挑战，保持经济平稳较快增长。面对跌宕起伏、错综复杂的经济形势，我们认真贯彻落实国家宏观调控政策和省委、省政府、市委的决策部署，坚定信心，主动应对，统筹兼顾，综合施治。上半年，重点抓了企业资金融通、煤电油运保障、控制物价和节能减排工作。成功举办了鲁南经济带临海产业区发展与金融支持推进会，签约金额达288亿元；成立了招商银行日照支行，日照商业银行在青岛设立分行获得批准。全市信用总量余额744.04亿元，比年初增加73.26亿元，其中各项贷款余额574.53亿元，增加46.16亿元。千方百计做好成品油、煤炭的调运、储备和配置工作，确保了企业生产和居民生活用电。全市规模以上工业实现增加值369.57亿元，增长28.2%，利税、利润分别增长25%和20%，增幅均居全省前列。通过增加成品粮油储备、实施临时价格干预、打击价格违法行为，有效抑制了物价上涨，全年居民消费价格总指数同比上涨4.8%，低于全国、全省平均水平。对节能减排实行增量和存量“双调整”，万元GDP能耗和规模以上工业万元增加值能耗均呈下降趋势，COD和SO2等主要污染物减排完成年度目标任务。年中，认真贯彻落实“稳增长、控物价，调结构、增效益，促民生、保稳定”的思路和措施，有效防止了经济大的起落。进入四季度以后，及时把工作重点转移到应对国际金融危机上来，抢抓国家和省扩大投资、拉动内需的机遇，按照中央关于“出手要快，出拳要重，措施要准，工作要实”的总要求，及时研究出台了23条政策措施，不失时机地连续报批重大项目，争取了工作上的主动。到年底，已有24个项目被列入全省重点建设项目，总投资达950.7亿元，分别占全省的10%和12%；争取中央新增投资项目13批、53个，总投资13亿元，其中中央补助资金1.4亿元，为经济平稳较快增长提供了有力支撑和保障。

（二）社会主义新农村建设扎实推进。农业综合生产能力进一步提高。粮食总产连续4年突破100万吨，去年达到106.65万吨，总产和单产分别比上年增加2.69万吨和10.6公斤。水产品总产达到46.47万吨，肉蛋奶产量增长6.1%，农机总动力增长4.96%。12家企业被列入第四批农业产业化省重点龙头企业，建成标准化生产基地41个，认证无公害产品和绿色食品100个。认真落实强农惠农政策，全年共发放小麦直补等各类补贴4.77亿元，免费为2000户农民发放了科技储粮小康仓。加大农村基础设施建设力度，新建农村公路350公里，农村客车通车率达到99.5%；建成了沭河莒县段综合治理一期、沭河橡胶坝、崮河及沙墩河治理工程，治理水土流失62平方公里，完成小型水库除险加固122座，新发展节水灌溉4.08万亩，开发治理土地9.2万亩；新建改造农村自来水工程120处，农村自来水普及率提高到88%，被省政府表彰为“村村通自来水工作先进市”；建成标准化供电村337个；新建农村户用沼气池2430个；创办农村社区服务中心61处，建成乡村综合超市269处。坚持把发展壮大县域经济作为统筹城乡发展、解决“三农”问题、建设新农村的重要载体，落实激励政策，加大扶持力度，县域生产总值、地方财政收入分别增长17%和31.8%。

（三）招商引资和大项目建设取得新突破。成功举办了2008国际海滨旅游城市发展峰会暨千家旅行社会盟、首届中国日照国际工艺品博览会、第三届中国日照国际物流高层论坛等大型活动，开展了赴中东、欧美、澳洲、日韩、新马、台湾等大型招商推介活动，使我市在提高对外知名度、拓展对外市场、扩大交流合作和招商引资等方面都取得了新的突破。去年全市共招引投资额1000万元以上的项目308个，实际到位资金276亿元，其中到账外资5.13亿美元，增长1.8倍，总额居全省第五位，总投资5亿美元的森达美棕榈油项目落户我市。成功举办了2008青岛日照合作峰会暨经贸洽谈会，签署了两市区域一体化发展和先进制造业、现代服务业、旅游文化合作、人才服务等一批协议，签约项目77个，计划投资总额104.79亿元，利用市外资金98.45亿元。大项目建设成效显著，华能日照电厂2×68万千瓦机组建成投运，威亚发动机二期年产30万台发动机项目、亚太森博二期年产100万吨木浆项目、青啤年产30万千升啤酒扩建工程以及中纺粮油、新昌食品、黄海粮油二期、华海造船、锦湖金马化工等项目顺利实施。334省道五莲至胶南段、335省道陈疃至三庄段、342省道岚山港至204国道段改扩建工程建成通车，后村220千伏输变电工程投入运行，日照港疏港高速公路、335省道三庄至莒县段开工建设。特别令人振奋的是，一批事关日照长远发展的大项目争取工作实现了重大突破：山钢与日钢重组顺利推进，大型钢铁精品基地建设前期工作迈出重要一步；鲁南临海产业区被省委、省政府列为省级综合配套改革试验区；全省“四纵四横”铁路网建设涉及日照的有“两纵两横”，我国南北沿海铁路大通道青岛至日照段今年有望开工建设，晋中南煤炭外运铁路大通道出海口正式确定在日照，日照保税物流中心（B型）获得国家批准设立。这些重大项目的突破，将为我市长远发展打下坚实的基础，同时凸显了日照在全省发展大格局特别是鲁南经济带中的重要战略地位，展现出日照更为广阔的发展前景，极大地鼓舞着全市人民以更加坚定的信心和决心，去创造日照美好的明天！

（四）特色经济和服务业发展迈出新步伐。日照港总体规划通过交通运输部审查，综合性大港和国家重要枢纽港的地位进一步确立。港口基础设施建设扎实推进，投资30亿元新建续建了石臼港区矿石码头二期、西区三期及岚北港区30万吨级油码头等重点工程。积极拓展港口物流功能，在岚山设立了全国第二个、我省第一个国家级木材贸易加工示范区。积极开拓腹地货源市场，与青岛港在临沂联合设立了国际集装箱合作箱站，赴山西、新疆开展港口推介系列活动，与乌鲁木齐市和伊犁自治州建立了友好城市关系，与阿拉山口口岸、霍尔果斯口岸及新疆八一钢铁、山西焦煤等重要通关口岸和货源单位建立了战略合作关系。全年完成港口货物吞吐量1.51亿吨、集装箱运量70.86万标箱，分别增长15.6%和64.7%。旅游经济进一步发展，全年共接待境内外游客1466万人次，实现旅游总收入82亿元，分别增长18.4%和24.3%。首届中日韩国际大帆船比赛和“日照”号单人无动力帆船环球航海取得圆满成功，日照游泳馆开工建设。大力扶持发展服务业，安东卫国际水产城、韩城摩尔新世界等大型商业设施建成使用，日照高新技术产业开发区被认定为全省首批12个“国际服务外包示范基地”之一。

（五）城市规划建设管理水平进一步提高。着眼于打造具有日照特色的国际水准滨海名城，借助国内外著名规划机构和业内专家，高起点编制了阳光海岸概念规划、北部旅游度假区概念规划、市级文化中心规划、临海产业区规划和综合配套改革试点方案，启动了城市中心区规划设计国际咨询招标，水上运动基地荣获“中国建设工程鲁班奖”。大力推进城建重点工程建设，完成了山东路拓宽改造和临沂路贯通工程，启动了阳光海岸整治建设工程，开展了15处村居改造工作，胶州至日照天然气长输管线日照段已全线贯通并具备接气条件。新增城市绿化面积300万平方米，海滨生态绿化项目被建设部推荐申报“2008年迪拜国际改善人居环境最佳范例奖”，我市荣获“中国人居环境奖”。加大城乡环境综合整治力度，对连云港路等14条城市道路、10条小街小巷和旧居住区进行集中整治，实施了青岛路、兴海路等5项防汛工程，推进了垃圾处理厂二期、城市污水再生利用二期工程建设，城市环境综合整治定量考核获全省第一。城建管理指挥中心和城管监督中心建成启用，规划批后监管得到加强，城市管理水平进一步提高。

（六）积极做好支援抗震救灾和青岛奥帆赛工作。广泛开展“为抗震救灾献爱心、为发展稳定做贡献”活动，举全市之力支援灾区抗震救灾和恢复重建，累计捐赠款物2.5亿元，总量居全省第6位，人均居第2位，无偿献血120万毫升，献血预约登记4540人。积极配合全国妇联、全国少年儿童基金会做好灾区学生接收安置工作，日钢安康家园共接收安置灾区学生20批、665人。圆满完成了对口援建北川县桃龙乡和盐亭县过渡安置房建造安装任务，在全省率先完成对口支援桃龙乡农户永久性住房修复和重建任务，乡驻地灾后恢复重建项目顺利开工，成为全省对口援建北川西部10乡镇中最早开工的市。在抗震救灾中涌现出莒县农民救灾志愿队、市公安消防救援队、卫生防疫监督队、公路抢通保通突击队、过渡安置房安装队、援川前线指挥部等一批先进群体和先模人物，受到中央和省、市表彰。全力支援青岛做好浒苔清理和奥运安保工作，为奥帆赛和残奥帆赛成功举办做出了积极贡献。历史将深刻铭记，支援抗震救灾和青岛奥帆赛，进一步展现出日照人民无私奉献的崇高精神和时代风采，我们为此而感到由衷的骄傲和无比的自豪！

（七）和谐社会建设深入推进。深入开展民生体系建设调查研究，扎实推进12件为民办实事重点项目，努力为群众解决就业、保障、住房、教育、医疗等方面的实际困难，去年市财政用于改善民生的投入达26.1亿元，比上年增长26%。全年新增城镇就业4.28万人，有组织转移农村劳动力2.3万人，对城镇零就业家庭、农村零转移就业贫困家庭做到了“动态消零”，高校毕业生就业率高于全省平均水平。全市新增社会保险参保人员7.3万人，企业退休人员待遇提高12%；先后两次提高城乡低保补助标准，城市低保标准由每人每月210元提高到235元，农村低保标准由每人每年800元提高到900元。有704户购房困难家庭获得年度经济适用房补贴资格，为1310户住房困难家庭发放了廉租房补贴，配套建设廉租房200套。全市农村义务教育债务化解完成60%，维修改造农村中小学校舍15.8万平方米，改造市区薄弱学校4所，全部免除了义务教育阶段学生杂费，免费为农村义务教育阶段学生提供教科书，为4000多名农村寄宿学生补助了生活费。全面启动城镇居民基本医疗保险，新农合医疗补助标准由40元提高到80元。积极组织实施“暖冬行动”，救助各类困难群体家庭7800户。免费实施白内障手术2067例，为300户农村贫困残疾人家庭进行了危房改造，向残疾人捐助轮椅800辆。积极改善居民出行条件，主动降低了公交车票价。切实加强农产品、食品、药品安全管理，质检中心、动物疫病检测中心建成启用。及时做好手足口病防治和“问题奶粉”处置等工作，维护了群众的健康权益。积极开展公共安全体系建设调查研究。进一步加强安全生产监督管理，全年安全事故起数下降31.1%；不断深化“五五”普法教育，健全完善“一警多能、合成作战、快速反应”的新型警务体系和应急预防体系；深入开展严打整治斗争，创建“社会治安综合治理优秀市”通过中央综治委验收；市级应急指挥平台建成启用，处置突发事件和防灾减灾水平进一步提高；认真做好信访工作，主动化解社会矛盾，全市保持了安定和谐的良好局面。

（八）精神文明建设和各项社会事业协调发展。大力加强社会主义精神文明建设，被授予“省级创建文明城市先进市”。依法深入开展全民国防教育，完善国防动员机制，国防后备力量建设得到加强。深入开展双拥共建工作，全面落实优抚安置政策，实现了全国双拥模范城“三连冠”。社区建设力度加大，新建城市社区18个，农村社区建设逐步推开。日照职业技术学院晋级为国家示范性高职院校，日照高级技工学校获准设立，莒县职业教育中心被评为国家级重点职业学校。成功举办了北京大学日照发展高层咨询会并开始进行实质性合作，与中国石油大学、山东大学等高校建立了市校全面合作关系，清大华创日照科技企业孵化器投入运行。高新技术产业进一步发展，全年实现产值220亿元，比上年增长60%，增幅居全省首位。文化卫生体育事业进一步发展。全市有50%的乡镇建成了综合文化站，30%的村居建成了文化大院， 80%的行政村实现了每月放映一场公益电影。为14处乡镇卫生院配备了X光机、B超等设备，建成规范化村卫生室227处，市区社区卫生服务人口覆盖率达到80%。我市运动员在北京残奥会上喜获铜牌，实现了奥运奖牌零的突破，日照被授予“振兴山东残疾人体育突出贡献奖”。新闻出版、广播电视、妇女儿童、外事侨务、对台事务、民族宗教、老龄、档案、史志、人防、气象、防震、打私、驻外机构、红十字会等工作都取得了新的成绩。计划生育工作得到加强，人口自然增长率控制在3.77‰，并获得全省一等奖。土地、矿产、海洋、森林、水资源节约利用成效明显，生态市建设扎实推进，我市被命名为“国家可持续发展先进示范区”。

（九）政府自身建设进一步加强。出台了《关于进一步加强自身建设的意见》，修订完善了《市政府工作规则》。在政府系统深入开展“学先进、找差距、促发展”解放思想大讨论，进一步提升了思想境界和工作标杆，促进了科学发展。全面落实政府信息公开、重大社会公共事项决策听证等制度，通过发放《2008日照市民生手册》，将政府公共服务项目、办事程序、办理时限等向群众公开，提高了政府工作的透明度，强化了监督机制。自觉坚持依法行政，主动接受市人大及其常委会的法律监督和市政协的民主监督，全年办理人大代表建议32件、政协委员提案166件，办复率、满意和基本满意率均达到100%。进一步畅通民意诉求渠道，建立快速反应机制，解决了一批事关群众切身利益和社会关注的问题。对现有行政许可项目和非行政许可审批项目进行了新一轮清理，共精简行政审批项目266项，精简比例35.42%；废止市政府规范性文件42件。在全省率先推行企业注册审批“一表通”，建立招商引资代办服务制度，进一步提高了办事效率和服务质量。全面落实党风廉政建设责任制，强化行政监察和审计监督，从严查处违法违纪案件，形成了勤政廉洁的政务环境。

各位代表！

过去的一年我们经历考验，克服困难，进一步巩固和发展了历届市领导班子和全市人民开创的良好局面，实现了经济平稳较快发展、社会和谐稳定、人民安居乐业。在历史罕见的挑战和困难面前，我们能够取得这些成绩，确实来之不易。这是省委、省政府和市委正确领导的结果，是市人大、市政协和社会各界监督支持的结果，是全市人民团结实干的结果。不平凡的一年展现了日照人民不平凡的精神，复杂多变的形势砺炼了日照人民勇往直前的气概。过去一年的实践再次表明，我们的干部队伍是一支精良的干部队伍，全市人民群众是英勇善战的人民群众！在此，我代表市人民政府，向辛勤工作在全市各行各业的广大劳动者和建设者，向给予我们监督支持的各级人大代表、政协委员，向各民主党派、工商联、无党派人士、各人民团体，向离退休老领导、老同志，向驻日照人民解放军和武警官兵，向所有关心支持日照建设的各界人士和海内外朋友，表示崇高的敬意和衷心的感谢！

在肯定成绩的同时，我们也清醒地认识到存在的困难和问题，特别是这次国际金融危机对我市工业生产、进出口贸易、港口运输、房地产市场、财政收入和城乡就业带来了冲击，保持经济社会又好又快发展的压力加大。与此同时，我市发展中的薄弱环节和矛盾问题依然存在，经济总量仍然偏小，结构层次偏低，增长方式比较粗放，节能减排形势严峻；对内对外开放力度有待进一步加大，广度有待进一步拓宽；县域经济仍然较为薄弱，县乡财政比较困难；影响社会和谐的因素和安全生产隐患不容忽视，部分群众在生活、就业、就医、社保、住房等方面还存在许多实际困难；政府个别部门和单位服务意识不强，办事效率不高，经济发展环境还需进一步改善。对这些困难、矛盾和问题，我们必须更加高度重视，采取更加有力措施，在新的一年里认真加以解决。

二、2009年政府工作任务

2009年是新中国成立60周年、地级日照市建立20周年，也是推进“十一五”规划顺利实施的关键一年。做好今年的工作意义重大，影响深远。综合分析今年的形势，可以说是困难矛盾与有利条件同在，严峻挑战与历史机遇交织，困难与挑战之中蕴含着重大机遇。战胜金融危机的冲击，实现既定的任务目标，首要的在于认清克服困难的有利条件，坚定必胜的信心。从宏观形势分析，我国仍处在重要战略机遇期，经济发展的长期趋势不会因为这场金融危机而发生根本性逆转。特别是中央适时对宏观经济政策作出重大调整，出台了一系列扩大内需的政策措施，省委、省政府及时对接落实中央的决策部署，必将有力地提振经济景气状况，同时为我市争取项目、资金和政策支持创造了难得机遇。从我市来看，省委、省政府继续实施“一体两翼”和海洋经济发展战略，在钢铁精品基地规划建设、鲁南临海产业区综改试点以及港口、铁路、公路等重大基础设施建设方面对我市给予倾斜和支持，为我市增强发展后劲、调整经济结构、转变发展方式、优化发展环境创造了千载难逢的机遇。经过改革开放30年、建市20年来的努力，我市基础设施承载能力不断增强，临港工业框架已经拉起，港口、旅游、体育、大学、海洋等特色经济蓬勃发展，主要经济指标增幅位居全省前列，加之能源和原材料相对充足，劳动力资源丰富，支撑经济发展的基础比较牢固。其实，危机也是一把“双刃剑”，国际金融危机本身也潜藏着重大机遇。历史经验告诉我们，每一次经济危机都会带来一次重新洗牌，每一轮经济收缩都孕育着新一轮的经济扩张。如果我们用好这次危机提供的“倒逼机制”和“洗牌机制”，加快提升产业结构层次，努力增创发展新优势，就有可能在区域经济竞争的赛车场上实现“弯道超越”、后来居上。

信心比黄金更重要，有信心才能有作为。在前所未有的困难和历史罕见的冲击面前，我们既要充分估计困难的严峻性，保持清醒的头脑，更要善于从危机中发现机遇，增强战胜困难的勇气和信心。我们有党中央、国务院和省委、省政府的正确领导，有建市20年来积累的成功经验和物质基础，有全市人民贯彻落实科学发展观、推动跨越发展的高涨热情，只要全市上下万众一心，负重奋进，扎实苦干，就一定能够经受住国际金融危机冲击的考验，在跨越发展的道路上阔步前进！

今年政府工作总的要求是：坚持以科学发展观统领全局，深入贯彻党的十七大、十七届三中全会和中央、全省经济工作会议精神，以开展深入学习实践科学发展观活动为动力，以积极作为、科学务实、坚定信心、变中求进为基调，以抓住新机遇、增创新优势、实现新跨越为主线，以保持经济平稳较快增长为首要任务，深入实施“四大战略”和“双轮驱动”，突出抓好现代产业、民生、公共安全三大体系建设，努力增创港口发展、临港工业、特色产业、城市可持续发展、县域经济、社会建设“六个新优势”，着力推动综合实力、发展方式、产业体系、文化建设、城乡统筹“五个新跨越”，加快推进全面建设小康社会进程，为建设经济文化强省做出新的贡献。

根据国内外经济形势的发展变化和省委、省政府的部署要求，综合考虑我市的各种因素和条件，今年全市经济社会发展的主要预期目标是：生产总值增长12%，地方财政收入增长11%，规模以上固定资产投资增长20%，社会消费品零售总额增长16%，规模以上工业增加值增长11%，进出口总值增长5%，实际利用外资在优化结构的基础上保持适当规模，城镇居民人均可支配收入增长8%，农民人均纯收入增长7%。主要约束性目标是：万元GDP能耗降低9.37%，规模以上工业万元增加值能耗降低11.24%，SO2排放减少5.83%，COD排放降低1.45%，主要污染物减排完成“十一五”目标的80%以上，人口自然增长率控制在6‰以内。

实现今年经济社会发展的任务目标，必须把握以下指导原则：一是要着力在保增长上下功夫。坚持把扩大内需作为保增长的根本途径，把加快转变发展方式和调整经济结构作为保增长的主攻方向，把深化改革开放作为保增长的强大动力，把改善民生作为保增长的出发点和落脚点，努力保持经济平稳较快增长。二是要更加自觉地贯彻科学发展观。坚持扩大内需为主与稳定外需相结合，保持增长速度与提高质量效益相统一，推进结构升级与扶持就业创业相协调，落实积极的财政政策和适度宽松的货币政策与有效防范财政金融风险相适应，加强政府调控与发挥市场机制相促进，努力实现科学发展。三是要把握好当前与长远的关系。在解决好现实问题的同时，注意谋划新的发展思路和长远发展战略，既促进当前经济平稳较快增长，又为今后发展积蓄后劲、打好基础。

今年要重点做好以下六个方面的工作：

（一）全面落实国家、省扩大内需的政策措施，努力保持国民经济平稳较快增长。充分利用国家实施积极财政政策和适度宽松货币政策的有利时机，全力以赴抓投资、促消费、扩内需、保增长。

一是保持投资的强劲增势。围绕国家和省重点支持的投资领域，继续组织争引和加快启动实施一批重点建设项目，突出争引和建设以港口、铁路、高速公路、能源、城市建设为重点的基础设施项目，以保障性住房、农村基础设施、医疗卫生、教育、文化、社会福利和促进就业为重点的民生工程项目，以科技创新、节能减排和生态环境工程为重点的产业结构调整项目。全力推进58项在建重点项目建设，开工建设坪岚铁路复线电气化改造、长春至深圳高速公路日照段等重大基础设施工程，力争开工建设黄岛至日照铁路，继续抓好新菏兖日铁路电气化改造、疏港高速公路、335省道三庄至莒县段改扩建等工程，进一步做好日照至潍坊高速公路、岚山至临沂高速公路、巨峰站至石臼港区西区铁路连接线、342省道西段改扩建等项目的前期工作。按照建设一批、储备一批、规划一批的原则，搞好项目的接续和储备。对项目资金使用和工程建设实行全程监督，严防截留挪用、滞留不用和违法违纪行为的发生。

二是努力扩大消费需求。把扩大消费与改善民生结合起来，进一步提高居民特别是农民和城乡低收入群体收入，引导企业在提高效益的基础上逐步增加职工工资，完善统筹城乡的社会保障体系，增强居民的消费信心和能力，并引导其由预期消费转化为即期消费。深入实施“万村千乡”市场工程，完善农村新型经营服务体系，认真落实家电下乡补贴政策，积极扩大农机具购置，努力启动农村消费。全面落实中央和省、市关于支持居民住房消费的优惠政策，鼓励居民购买普通商品房，扶持启动农村住房建设。积极培育消费热点，鼓励居民增加汽车消费，扩大文化娱乐、体育健身、旅游休闲消费，推动消费结构上档升级。把发展服务业与扩大消费紧密结合起来，进一步完善政策措施，加快拓展生产性服务业，提升消费性服务业，培育现代服务业，推动服务业更好更快发展。

三是千方百计稳定外贸出口。引导外贸企业加快实施市场多元化战略，在稳定现有市场的基础上，积极开拓新兴经济体和发展中国家市场。大力实施科技兴贸战略，努力提高出口产品的科技含量和附加值。抓住国际市场价格走低的时机，增加关键设备进口，提高企业装备水平。支持企业实施“走出去”战略，积极稳妥地进行境外投资，建立境外资源开发基地，开展跨国经营。加大对出口企业的扶持力度，建立出口绿色通道，减免口岸收费，及时足额落实出口退税，对企业参加境外展览和市场考察给予补助。

四是加大对企业的支持保护力度。各部门要从各自职能出发，积极主动地为企业“加油松绑减压”，帮助企业渡过难关。在财政收支压力加大的情况下，年内安排1亿元工业发展专项资金，对企业给予贴息、补助和风险补偿。引导金融机构加大信贷投放力度，满足各类企业特别是中小企业的合理资金需求。继续举办银企合作交流会，加大异地金融机构争引力度，积极为银企合作牵线搭桥，降低企业融资成本。搞好3家小额贷款公司试点工作，逐步扩大覆盖面。引导企业运用上市、股权融资、并购重组、发行债券等方式集聚资金，对企业上市实行激励政策，年内力争有1—2家企业进入上市程序。加强中小企业信用担保体系建设，完善信用担保风险补偿机制，对市信用担保公司进行注资和增持，支持区县设立贷款担保机构，争取设立产业发展基金。严格控制和清理涉及企业的检查、评比、达标、表彰活动，凡无法律法规依据或中央和省文件无明确规定的一律取消。实行认定检查结果共享制，坚决纠正多头重复检查行为。全面清理整顿涉及企业的各种收费，对国家和省明令取消或停止征收的行政事业性收费要坚决停止，对保留的收费项目一律执行最低标准，尽快清理取消一批市级或部门收费项目，在日照经济开发区、山海天旅游度假区、日照高新技术产业开发区实行“一口收费”制度。在加大外部扶持的同时，引导企业眼睛向内抓管理，眼睛向外拓市场，团结一心渡难关，千方百计保增长。

我们坚信，只要认真贯彻落实好国家宏观调控政策，采取符合日照实际的有针对性的举措，团结一心，大干苦干，就一定能够实现国民经济的平稳较快增长，一定能够在金融危机的逆境中实现新的发展和新的跨越！

（二）以党的十七届三中全会精神为指导，深入推进农村改革发展。在经济发展遭遇外部冲击的情况下，稳定农业农村形势显得尤为重要。要更加重视做好“三农”工作，把增加农民收入作为扩大内需的重要内容，切实保证农业增产、农民增收、农村稳定。

一是努力提高农业综合生产能力。把确保粮食安全放在首位，坚守基本农田、耕地面积、粮食总产“三条底线”，突出抓好莒中平原、莲北平原等粮食主产区建设，建成小麦高产示范区5万亩，确保全市粮食总产稳定在100万吨。因地制宜推进农业结构调整，优化种植业，壮大畜牧业，提升水产业，抓好林果业，开发旅游观光农业，年内新增茶园1.5万亩、瓜菜1万亩。加快推进农业产业化经营、标准化生产，培植壮大农业龙头企业群体，鼓励发展各类专业合作组织。加大科技兴农力度，搭建新型农业科技创新体系和推广服务网络，为现代农业发展提供科技支撑。

二是千方百计增加农民收入。认真落实中央、省各项强农惠农政策，全面执行粮食最低收购价，落实国家提高农资综合直补、良种补贴、购买农机具补贴标准政策，确保每项补贴都不折不扣地落实到农民手中。高度重视农民工就业出现的新情况，进一步加大职业技能培训和有组织转移力度，拓展农村劳动力就业渠道。加强农村小城镇和特色产业园区建设，为农民务工经商提供载体。加快农村经济结构调整，大力发展以农副产品加工和农村服务业为基本内容的农村二三产业，提高农民非农收入。

三是加强农村基础设施建设。开工建设小仕阳水库、长城岭水库和110座小型水库除险加固工程以及沭河莒县段综合治理二期、青峰岭水库灌区续建配套和节水改造等重点水利工程，搞好龙潭沟水库规划建设，开发治理土地10万亩，治理水土流失56.7平方公里，新增造林面积6万亩。建设招贤、秦楼220千伏，虎山、碑廓、汪家台110千伏输变电工程，建成8个电气化乡镇、413个电气化村；改建农村公路200公里；新增农村沼气用户4800户；建设村村通自来水工程100处，使农村自来水普及率提高到90%以上。

四是继续深化农村改革。认真贯彻党的十七届三中全会精神，赋予农民更加充分和有保障的土地承包经营权，允许农民按照依法自愿有偿的原则发展多种形式的土地规模经营。土地承包经营权流转不得改变土地集体所有性质，不得改变土地用途，不得损害农民土地承包权益。坚持有计划、分阶段、分批次地推进新农村建设，在条件较好的地方率先培植起各具特色的新农村建设典型，逐步形成特色突出、全面发展的城乡格局。加大对农业的支持保护力度，确保各级财政对农业投入增长幅度高于经常性收入增长幅度。积极推进农村金融体制改革，争取设立农村资金互助社、小额贷款公司、村镇银行等新型农村金融机构。建立引导信贷资金和社会资金投向农村的激励机制，对涉农贷款给予税收减免或费用补贴。落实政策性农业保险制度，鼓励商业性保险机构开展农业保险业务。积极探索农村集体经济的有效实现形式，增强村级积累和发展能力。深化集体林权和小型水利设施管护体制改革。深入推进乡镇机构改革，转变乡镇政府职能。经过努力，全市“三农”工作在新的一年一定会取得新的进步，社会主义新农村建设一定会揭开新的一页！

（三）抓好现代产业体系建设，推动经济结构优化升级。按照形成财源经济、环保经济、节能经济、特色经济以及循环经济的思路，通过存量调整、增量优化、政策引导、合理布局，加快构建以高新技术产业为先导，临港工业、先进制造业和综合旅游业为支撑，包括港口现代物流业在内的服务业全面协调发展的现代产业体系。

一是统筹搞好现代产业体系规划。依据现有条件、所面临的新的重大机遇与发展优势，综合考虑临海产业区、综合改革试验区规划，搞好钢铁精品基地、重化工基地及其产业链项目规划和布局规划，统筹谋划和合理布局高新技术产业、临港工业、先进制造业、综合旅游业、物流业及其他服务业、文化体育医疗等各项社会事业项目以及农村各产业的发展，使三次产业更加协调，主导和优势产业更加突出，特色产业更加鲜明。把产业发展规划与城市建设和新农村建设结合起来，与港口、铁路、高速公路等重大基础设施发展结合起来，与打造滨海名城结合起来，协调推进，配套实施。

二是增创工业发展新优势。继续组织实施百项重点工业项目，突出抓好日照钢铁精品基地资源重组、土地征用、村庄搬迁以及生活办公服务区建设等配套服务工作，为项目尽快开工创造有利条件。加快推进亚太森博二期、威亚发动机二期、日广水泥和黄海粮油二期等一批重大项目建设，围绕主导优势产业谋划协作配套产业发展，拉长产业链，推动产业集群化发展。修订完善鼓励县域工业发展的政策措施，支持各区县立足港口优势和地方资源优势大力引进临港工业配套型、产业协作型、农产品加工带动型、财税增收型项目，推动县域经济跨越发展。

三是全面提升港口经济发展水平。按照“港城一体、港带一体、港桥一体、陆海一体”的要求，高标准实施新一轮港口总体规划，年内投资20亿元新建续建13项港口重点工程，建成石臼港区木片码头续建工程、岚山港区液体化工1号2号码头、岚桥两个5000吨级液体化工泊位、童海港业码头扩建等项目，加快推进石臼港区西区三期、防波堤和岚北港区30万吨级油码头等重点在建项目，争取开工建设岚北港区两个30万吨级矿石码头、童海港业两个5000吨级液体化工泊位等重点工程。建成鲁南日照国际贸易与航运服务中心，全面启动电子口岸建设，进一步优化口岸环境。加快保税物流中心建设，争取早日封关运作。继续加大腹地货源市场开拓力度，加强与青岛港、中远集团等大企业的合作，推动集装箱业务快速发展。全年完成港口货物吞吐量1.58亿吨，力争1.62亿吨，集装箱运量80万标箱，力争90万标箱。

四是大力繁荣发展服务业。发挥好省、市服务业发展引导资金的作用，加大对“三大载体、八大产业”的扶持力度，加快培植临港物流园、安东卫国际水产城、龙鼎花生电子商务等重点园区、企业和市场，做大做强一批服务业项目。抢抓国际服务外包转移机遇，加快发展服务外包产业。坚持打造四季游、休闲度假游和高档精品游三大理念，制定促进旅游业发展的政策措施，加大旅游资源保护整合开发力度，理顺旅游管理体制，鼓励支持重点旅游企业发展。加快推进室内滑雪溜冰大型综合游乐项目、阳光海岸、太阳城旅游度假区、涛雒水乡古镇等项目建设。继续开展融招商、会展、城市推介、旅游展销等为一体的大型综合活动，加大客源市场开拓力度，年内旅游人数力争突破1700万人次，实现旅游收入100亿元。

五是加快发展高新技术产业。进一步加强高新技术产业开发区、大学科技园和科技孵化器等载体建设，深入推进与北京大学、山东大学、中国石油大学等知名院校的产学研合作以及与中国建材集团等“中字号”大企业的合作，围绕电子信息、生物医药、精细化工、新材料、新能源、海洋高新技术等领域组织实施10个高新技术重大专项。进一步加大人才培养、引进、使用力度，制定和完善引进人才特别是人才带项目的激励政策，努力形成人才聚集效应。认真落实国家扶持高新技术产业发展的政策措施，加大对企业创新支持力度，对新认定的市级以上企业技术中心和名牌产品给予奖励，年内建设市级以上工程技术研究中心10个，完成规模以上工业企业高新技术产业产值280亿元以上。

六是坚持增量和存量“双调整”，继续抓好节能减排工作。进一步强化对重点区域、重点行业和重点企业的能耗、水耗、污染物排放控制，实现主要产品能耗、重点工序能耗持续下降。坚持从源头抓起，控制“两高”项目建设。逐步淘汰落后产能，年内淘汰立窑水泥生产线7条、关停钢铁转炉和高炉各两座。加大工程减排、结构减排和管理减排力度，确保减排项目按期建成投运、稳定达标排放。进一步完善责任体系，落实“一票否决”和限批等措施。

（四）深入实施对外开放和改革创新“双轮驱动”，增强经济社会发展后劲与活力。抓住鲁南临海产业区列入省级综合配套改革试验区和钢铁精品基地在我市布局等多个重大机遇，进一步加大“走出去”、“请进来”力度，年内重点组织好赴韩日、欧美、新加坡、港台以及沪粤江浙等投资环境推介与招商引资活动，办好第二届中国日照国际工艺品博览会、投资基金与产业对话会、海内外知名企业家“齐鲁行”等大型系列经贸活动，为招商引资搭建平台。围绕高新技术产业、现代服务业、先进制造业、环保产业和基础设施等领域，积极争引世界500强、“中字号”、“民字号”大企业等旗舰型项目、龙头型项目和产业集群项目。对去年签订的招商引资项目要明确责任，抓住不放，逐项落实。积极推进现代商用车、森博浆纸三期高档文化用纸等重点项目，加快光伏太阳能、森达美棕榈油、苏米特LED项目建设，力争早日建成投产。建立招商引资项目税收、财力贡献评价机制，大力发展总部经济，提高招商引资实效。加快打造有利于企业发展的技术创新平台、人才支撑平台、投融资平台和生产服务平台，进一步营造重商、亲商、扶商的氛围，增强投资者的信心。

认真落实青岛日照合作峰会暨经贸洽谈会取得的成果，全面推进两市在发展规划、市场体系、产业发展、对外开放、创新体系、基础设施、生态环保、文化建设、社会管理九个方面的“一体化”进程。联合成立区域发展总体规划和各类专项规划编制机构，尽快启动规划制定、政策研究等工作。加快对接青岛现代产业体系战略布局，重点围绕电子信息、汽车机车船舶、石油化工、海洋科技、软件开发、现代服务业等优势产业集群，不断深化两市产业和企业间的合作。联手推进黄岛至日照铁路建设，积极争取建设青岛至日照城际铁路，打造“一小时经济圈”，营造“同城效应”。

以鲁南临海产业区综合配套改革试点为契机，在对外开放、企业改革、土地管理、城乡统筹、公共服务、循环经济、资源节约等方面大胆探索创新，为面上的改革积累经验。适时跟进国家出台的重大改革措施，全面实施增值税转型改革，认真做好燃油税费改革相关配套工作，建立健全矿产资源有偿使用和生态环境补偿机制，加快推进医药卫生体制改革，积极稳妥地搞好政府机构改革。进一步完善国有资产监管方式，建立科学的业绩考核、综合评价和责任追究体系，继续推动城市公用设施等国有资产的资本化运作。进一步优化外部环境，鼓励、支持和引导非公有制经济健康发展。把支持企业发展、培植财源与加强税收征管结合起来，努力挖掘增收潜力，保持财政收入稳定增长。

（五）提升城市规划建设管理水平，打造具有日照特色的国际水准滨海名城。充分发挥我市的生态、资源、后发优势，坚持高起点规划、大手笔建设、精细化管理，着力完善城市功能，优化空间布局，提高城市品位，积极争创“联合国人居奖”。

一是继续提高城市规划水平。加快城市中心区规划设计国际咨询招标工作，引进国际知名城市规划和设计团队，按照高档、高层、精品的原则，高起点、高标准完成城市中心区设计并起步建设，着力把城市中心区打造成能够满足国际商务、旅游、休闲、体育等需求，并体现日照特色的高档中央商务区。按照“双城一区、轴向拓展、陆海一体、东西联动”的要求，完善临海产业园区、涛雒巨峰组团、两城河山组团及北部旅游度假区组团规划，着手编制市区公共艺术规划、南部海岸带控制规划。完成日照城乡一体化规划编制，形成城乡统筹、相互衔接、全面覆盖的城乡规划体系。加强规划批后监管，维护规划的权威性和严肃性。

二是抓好城市重点工程建设。实施阳光海岸整治工程，保护和恢复沙滩、礁石、湿地等原始生态环境，建设一批园路、广场、雕塑及景观小品，配套建设必要的旅游服务设施，沿阳光海岸建设一条景观优美的旅游观光大道，打造中国沿海独树一帜的阳光海岸带。进一步完善奥林匹克水上公园的功能，抓好游泳馆、城市规划展览馆及水上运动基地续建项目等精品工程，不断丰富我市水上运动之都、水上休闲之都的内涵。坚持“森林式”与“花园式”相结合，提升城市绿化水平，年内新增城市绿化面积272万平方米。实施204国道改线和北海路建设工程，建设改造淄博路、菏泽路等41条城市道路。争取开工建设日照水库向市区供水三期工程，新增日供水能力10万吨。

三是不断提升城市形象和文明层次。深入推进城乡环境综合整治，突出十一届全运会比赛场馆、城乡结合部、城市出入口、旧住宅区、小街小巷及老城区防洪设施等重点部位，完善供水、排水、供热、燃气管网，加快垃圾处理厂配套工程及污水再生利用二期工程建设，实施市区张家河、香店河等5条河道治理工程，继续推进祥博社区等15个旧村改造。牢固树立人性化、规范化、法制化的管理理念，不断提升城市管理水平。同时，在广大市民中进一步营造热爱日照、建设日照、提升日照文明层次、维护日照美好形象的浓厚氛围，用我们的双手，着力打造具有日照特色的国际水准滨海名城，把我们的家园建设得更加美好！

（六）加快构建民生体系和公共安全体系，扎实推进和谐社会建设。坚持把民生体系和公共安全体系建设作为系统工程来推进，整合各方力量，构建长效机制，努力保持日照长治久安、人民安居乐业。

一是千方百计扩大就业。今年的就业形势十分严峻，必须实行更加积极的就业政策，调动各方力量促进就业再就业。把大学生就业放在突出位置，鼓励用人单位积极吸纳高校毕业生，鼓励毕业生到基层、农村和中小企业就业创业。引导企业履行社会责任，尽量不裁员或少裁员，防止出现大规模集中裁员现象。年内新增城镇就业2.9万人，下岗失业人员再就业8000人，有组织转移农村劳动力1.5万人，城镇登记失业率控制在3.5%以内。

二是进一步完善社会保障体系。加大社会保险扩面征缴力度，把更多的非公有制企业、个体工商户和灵活就业人员纳入社会保险覆盖范围。建立低保补助标准与物价变动相联系的动态调整机制，农村低保标准由900元提高到1000元。提高企业退休人员养老金，月人均增加130元左右。全面推进城镇居民基本医疗保险，年内参保率达到75%。提高新型农村合作医疗报销比例，简化服务程序，扩大参合农民受益面。进一步完善经济适用房货币直补和廉租房制度，配建廉租房300套，实现三条住房保障线的有效衔接。完善社会救助体系，积极筹建集社会福利院、儿童福利院、老年公寓、救助管理站、流浪未成年人救助保护中心于一体的社会福利中心。

三是扎实做好社会稳定工作。整合相关资源，积极推进公共安全体系建设。严格落实安全生产责任制，建立安全生产长效机制，坚决防止重特大安全事故发生。加强农产品质量检测和动植物疫病防治体系建设，建立从田间地头到加工生产车间、商品货架、家庭餐桌的一体化、链条式食品、药品、农产品“三品”安全监管模式。进一步完善应急信息平台建设，健全应急预案体系，有效应对自然灾害、事故灾难、公共卫生事件和社会安全事件。正确处理新形势下的人民内部矛盾，注重从源头上解决影响社会稳定的问题。深入推进“平安日照”建设，强化社会治安综合治理，严厉打击各种违法犯罪活动，切实增强人民群众的安全感。

四是加强精神文明建设，推动文化大发展大繁荣。大力弘扬爱国主义、集体主义、社会主义思想，加强责任心、诚心、爱心、孝心“四心教育”，搞好社会公德、职业道德、家庭美德、个人品德“四德建设”，开展“城乡文明牵手共建”活动，积极争创省级文明城市。扎实做好双拥共建和优抚安置工作，争创全国双拥模范城“四连冠”。突出抓好民兵预备役基层建设，提高国防动员建设质量。围绕建设“滨海文化名城”，着力做深“昨天的文化”、做实“今天的文化”、做活“明天的文化”，加快打造莒文化、龙山文化、海洋文化、生态文化、茶文化等特色文化品牌，培育动漫游戏、数字传媒等高端文化产业和创意产业，做大黑陶、农民画等传统优势文化产业。加快建设市级综合文化中心、文化艺术博览中心，启动建设市图书馆，年内力争所有乡镇全部建成综合文化站；全面完成有线电视“村村通”工程，实施市区有线电视数字化整体平移。

五是积极发展各项社会事业。落实教育优先发展战略，积极促进教育公平，实施农村中小学校舍维修改造、教学仪器更新、“两热一暖一改”和办学条件标准化工程，完善义务教育经费投入保障机制，适当提高农村中小学公用经费标准。加强公共卫生体系建设，改造建设市人民医院、市中医院病房楼等设施，推进19处市县级医疗卫生机构建设项目，完成村级卫生室改造547处、设备配置563处。高度重视残疾人工作，实施“五有惠残共享”工程。精心组织十一届全运会日照赛区比赛、2009中日韩国际大帆船比赛等重大赛事，做好2010年中国水上运动会筹备工作。积极发展新闻出版、妇女儿童、外事侨务、对台事务、民族宗教、老龄、档案、史志、人防、气象、防震等事业。严格落实人口目标责任制，积极推进计生优质服务，努力稳定低生育水平，提高人口素质。深化国土资源使用制度改革，保持耕地总量动态平衡。以迎接国家环保模范城复查为契机，进一步加大生态保护力度，让日照城乡天更蓝、水更清、空气更加怡人、人与自然更加和谐！

六是继续做好对口支援桃龙乡灾后恢复重建工作。年内完成对口援建乡驻地小学等7个项目。积极参与北川新县城和工业园区建设，为“再造一个新北川”做出应有的贡献。

三、大力加强政府自身建设

加强和改进政府自身建设，打造一心为民、务实高效、公正廉洁的政府，是时代的要求和人民的期盼。要以开展深入学习实践科学发展观活动为契机，按照“为民、务实、清廉”的要求，进一步加强政府作风建设，不断提高为民执政、为民服务的水平，努力建设人民满意的政府。

一是着力建设亲民政府。各级政府领导干部要切实增强亲民意识，做到在思想感情上亲近群众，在领导作风上贴近群众，在生产生活上关心帮助群众，在工作上依靠群众。要始终保持与人民群众的血肉联系，经常深入基层和群众，及时了解全市人民想什么、盼什么，支持什么、反对什么，努力使政府的各项工作更加符合客观实际和群众意愿。要切实转变领导作风，力戒官僚主义和形式主义，坚决不搞劳民伤财的形象工程，不搞违背民愿的长官意志，不搞急功近利的短期行为。要进一步拓宽民意诉求渠道，带着深厚的感情倾听和处理群众反映的困难与问题，凡符合政策规定的要立即解决，政策界定不明确的要创造条件解决，对虽不符合政策规定但属于特别困难群众反映的问题，也要通过社会救助等办法提供帮助。要高度关注下岗职工、老弱病残、鳏寡孤独、未就业毕业生及城乡低保对象等弱势群体，及时帮助他们解决就业再就业、生活保障、子女上学、看病等方面的问题，让他们感受到党和政府的关怀与温暖。要精心组织实施2009年度为民办实事重点项目，对虽未列入实事项目但群众关注的问题，也要从发展生产、增加收入、改善环境等方面入手，合情合理地加以解决。

二是推动政府工作全面提速。各级政府和政府各部门要认真贯彻落实市政府新近出台的《关于进一步转变机关作风，提高行政效能，推动政府工作全面提速的意见》，全面推行岗位责任制、首问负责制、服务承诺制、限时办结制和失职追究等制度，简化办事程序，提高工作效率。要强化政府工作人员的大局意识、责任意识和服务意识，坚决杜绝“生、冷、硬、横、顶、拖”等衙门作风，坚决遏制和查处“不作为、慢作为、乱作为”行为。继续深化行政审批制度改革，对保留的审批事项进一步简化环节，规范流程，集中办理，缩短时限。年内建成网上审批和电子监察系统市级平台，实现行政审批事项网上办理和实时在线监察。要切实加大行政效能监察力度，对执行公务情况实行明察暗访，组织企业对部门服务情况进行评价，严格落实问责制度和奖惩措施。

三是严格依法行政。认真贯彻落实国务院《关于加强市县政府依法行政的决定》，坚持政府常务会议学法制度，严格依照法定权限和程序行使权力。建立健全公众参与政府决策的规则和程序，对涉及重大公共利益和群众切身利益的决策要向社会公开征求意见。加强对行政执法部门的监督管理，严格落实行政执法责任制、案件评查制、错案追究制、评议考核制和行政赔偿制，促进公正执法、文明执法。认真落实政务信息公开制度，不断拓展公开的领域和内容，切实保障群众的知情权、参与权、表达权和监督权。全面推进城乡社区建设，完善群众自治制度，不断扩大基层民主。各级政府要自觉接受人大及其常委会的法律监督和政协的民主监督，广泛听取民主党派、工商联、无党派人士和各人民团体的建议意见，主动接受社会舆论和人民群众的监督，确保权力在阳光下运行。

四是坚持廉洁从政。认真落实党风廉政建设责任制，切实纠正部门和行业不正之风，严厉查处各类违法违纪案件。不断完善建设工程招投标、经营性土地招拍挂、政府采购等制度，建立廉政建设的长效机制。要保持和发扬艰苦奋斗的优良传统，坚持勤俭办一切事业，在今年特殊困难的情况下，更要牢固树立过“紧日子”的思想，年内车辆费、会议费、接待费、出国费等支出一律实行零增长。各级领导干部要认真落实“一岗双责”，坚持分管工作和廉政建设“一肩挑”，关口前移，防微杜渐，切实把好自身廉洁关、分管部门干部队伍的防腐关、家人与身边工作人员的清廉关，老老实实做人，扎扎实实做事，虚心接受监督，以清正廉洁的实际行动，赢得全市人民的信赖与支持！

各位代表！

今年我们迎来了共和国60华诞，地级日照市也走过了20个春秋。回首往昔，勤劳智慧的日照人民创造了辉煌业绩，美丽的新兴港城正在迅速崛起；展望未来，日照的发展空间更加广阔，前景更加美好。尽管前面还有许多困难，但任何困难也难不住勇往直前的日照人民；尽管前面还有许多挑战，但任何挑战也挡不住日照跨越发展的步伐！让我们紧密团结在以胡锦涛同志为总书记的党中央周围，在中共日照市委的坚强领导下，同心同德，开拓进取，坚定信心，真抓实干，为实现富民强市新跨越而不懈努力，以优异成绩向建国60周年、建市20周年献礼！
